# Neurofilament markers in serum and cerebrospinal fluid of patients with amyotrophic lateral sclerosis

**DOI:** 10.1111/jcmm.17100

**Published:** 2021-12-06

**Authors:** Jiaying Shi, Xiaohui Qin, Xueli Chang, Hong Wang, Junhong Guo, Wei Zhang

**Affiliations:** ^1^ Department of Neurology First Hospital, Shanxi Medical University Taiyuan China; ^2^ Department of Encephalopathy Changzhi Hospital of Traditional Chinese Medicine Changzhi China

**Keywords:** amyotrophic lateral sclerosis, axonal damage, biomarker, neurofilament light chain, phosphorylated neurofilament heavy chain

## Abstract

This study aims to determine the serum and cerebrospinal fluid (CSF) levels of neurofilament light chain (NFL) and phosphorylated neurofilament heavy chain (pNFH) in amyotrophic lateral sclerosis (ALS) patients, and to explore their feasibility as valid biomarkers for quantifying disease progression and predicting individual prognosis. 52 patients with ALS and 30 controls with noninflammatory neurological diseases were included. NFL and pNFH levels in serum and CSF were measured by enzyme‐linked immunosorbent assay. Our findings showed that serum and CSF levels of NFL and pNFH in ALS patients were significantly increased. These values were negatively correlated with disease duration (except CSF NFL with disease duration) and ALSFRS‐r score, and positively correlated with disease progression rate (DPR) and upper motor neuron (UMN) score, but did not correlate with bilateral median and ulnar nerve compound muscle action potential (cMAP) amplitudes (except a weak correlation between CSF NFL and cMAP amplitudes). The optimal cut‐off values with high sensitivity and specificity were obtained in ROC curve analysis to discriminate ALS from controls. Kaplan‐Meier survival curves illustrated that survival was significantly shorter for patients with higher neurofilament levels at diagnosis. The Cox proportional hazards regressions confirmed that NFL and pNFH were significant predictors of survival. Overall, NFL and pNFH in serum and CSF can be used as reliable biomarkers in ALS.

## 1 INTRODUCTION

Amyotrophic lateral sclerosis (ALS) is a fatal neurodegenerative disorder due to selective motor neuron loss in the corticospinal tract, brain stem nuclei and ventral roots of the spinal cord.^1^ There remain limited therapeutic options to date with only two FDA‐approved drugs, riluzole and edaravone. Most of the patients have a poor prognosis and die from respiratory paralysis or pulmonary infections within 3–5 years of clinical onset.^1^ Hence, biomarkers are desperately needed for this devastating disease to identify therapeutic targets, stratify patients for clinical trial entry and serve as objective tools in subsequent trial access. Mounting evidence demonstrated that neurofilaments (NFs) levels, especially phosphorylated neurofilament heavy chain (pNFH) and neurofilament light chain (NFL), were elevated in serum and cerebrospinal fluid (CSF) of ALS patients.^2^, ^3^, ^4^, ^5^, ^6^, ^7^, ^8^, ^9^ NFs have been regarded as the most promising alternatives among the numerous candidate biomarkers for ALS. However, research on altered levels of NFs in Chinese ALS patients is relatively limited. This study aims to investigate the NFL and pNFH levels in the CSF and serum of ALS patients, analyse their clinical relevance to clinical parameters, and verify their roles as potential biomarkers in ALS.

## 2 MATERIALS AND METHODS

We recruited 52 patients diagnosed with ‘clinically definite’ or ‘clinically probable’ ALS and 30 controls with noninflammatory neurological disorders in this study. Demographic and clinical characteristics of patients were recorded. Disease duration was defined as the number of months between onset of symptoms and biofluid sample collection. The disease progression rate (DPR) was calculated as follows: (48−ALSFRS‐R score at the time of sampling)/disease duration (months). Survival duration was defined as time from symptom onset to permanent assisted ventilation (PAV) (≥22 h/d noninvasive ventilation), tracheostomy or death.

Blood and CSF samples were collected and then centrifuged at 4°C (blood: 1,500 *g*, 10 min; CSF: 800 rpm, 5 min). Supernatants were immediately frozen and stored at –80°C until assayed. The NFs concentrations were measured using commercial enzyme‐linked immunosorbent assays (ELISAs) in accordance with the manufacturer's instructions (pNFH; Abcam; NFL; Novus). The analytical sensitivity of the ELISA was 9.38 pg/ml for NFL and 20 pg/ml for pNFH. The mean intra‐assay coefficient of variation (CV) was below 10%, and the inter‐assay CV was below 12% for both NFL and pNFH concentrations.

Statistical analysis was performed using IBM SPSS Statistics SPSS (version 23.0) and GraphPad Prism 7. Continuous variables were tested for normal distribution using the Shapiro‐Wilk test. Normally distributed data were represented as the mean ±standard deviation and non‐normally distributed data as the median and interquartile range [IQR, P50 (P25, P75)]. The Student t test or the Mann‐Whitney U test was used to determine the significant differences between two groups for normally distributed data or non‐normally distributed data, respectively. A Pearson's correlation or Spearman's correlation test was performed for the correlation analysis. The area under the curve (AUC) and optimal cut‐off levels were calculated using receiver operating characteristic (ROC) curves. Two‐sided tests were conducted for all statistical analysis, and the level of statistical significance was set at *p* < 0.05. Kaplan‐Meier methods were adopted to estimate the survival of ALS patients and differences among groups were assessed with log‐rank test (Mantel‐Cox). Univariate Cox proportional hazards regressions were performed to identify predictors of the mortality hazard ratio (HR) in ALS patients and parameters with a p‐value below 0.1 were subsequently tested in a multivariate Cox model via the stepwise forward approach.

## 3 RESULTS

The demographic and clinical characteristics of the participants are displayed in Table 1 and Tables S1 and S2. Levels of serum NFL (S‐NFL), CSF‐NFL, serum pNFH (S‐pNFH) and CSF‐pNFH in the ALS group were significantly higher than in the control group (Figure S1). Among different subgroups based on median values, the serum and CSF NFs levels were higher in rapidly progressive group than in slowly progressive group (Table S3). In correlation analysis between NFs levels and ALS clinical parameters, there were no significant correlations between NFs levels and age, height, weight, gender or onset site (Table S4). The NFs concentrations were negatively correlated with ALSFRS‐r score and disease duration (except CSF NFL with disease duration, rho = −0.204, *p *= 0.227), and positively correlated with DPR and UMN score, but did not correlate with cMAP amplitudes (except a weak correlation between CSF NFL and cMAP amplitudes, rho = −0.361, *p *= 0.039) (Figures [Link], [Link], [Link]). In addition, positive correlation was evident between S‐NFL and CSF‐NFL (rho = 0.517, *p *= 0.001) as well as between S‐pNFH and CSF‐pNFH (rho = 0.809, *p *< 0.0001) (Figure S5). The optimal cut‐off values to discriminate between ALS and controls were calculated at 500 pg/ml S‐NFL (a sensitivity of 88.5% and specificity of 83.3%), 408 pg/ml S‐pNFH (a sensitivity of 100% and specificity of 83.3%), 647 pg/mL CSF‐NFL (a sensitivity of 97.3% and specificity of 96.7%) and 474 pg/ml CSF‐pNFH (a sensitivity of 97.8% and specificity of 97.2%) in ROC curves (Figure S6).

**TABLE 1:** Demographic characteristics of patients with ALS and controls

	ALS (serum)	Control (serum)	ALS (CSF)	Control (CSF)
Sex (Male/ Female)	30/22	15/15	23/14	15/15
Age (years)	59.17 ± 11.28	56.13 ± 6.08	60.27 ± 10.45	56.13 ± 6.08
Height (cm)	165.88 ± 8.06	167.43 ± 7.60	166.27 ± 7.96	167.43 ± 7.60
Weight (kg)	58.95 ± 9.73	62.87 ± 10.88	59.00 ± 9.51	62.87 ± 10.88
Disease duration (months)	8.0 (5.0–12.8)	NA	8.0 (6.0–13.0)	NA
Site of onset (Bulbar/Spinal)	16/36	NA	13/24	NA
ALSFRS‐r	37.5 (30.0–41.0)	NA	38.0 (30.0–41.5)	NA
UMN score	5.0 (3.0–11.0)	NA	5.0 (3.0–10.5)	NA
DRP	1.44 (0.61–2.69)	NA	0.80 (0.57–2.35)	NA
NFL (pg/ml)	649.56 (569.12–835.61)*	358.4 (300.03–441.23)	2460.84 (2204.56–2707.11)*	328.49 (318.61–348.73)
pNFH (pg/ml)	615.23 (517.68–723.60)*	237.25 (206.00–375.75)	1952.34 (1498.08–2452.69)*	222.23 (147.45–265.75)

Note

Data are presented as n, mean ±SD, or median (interquartile range).

Abbreviations: ALS, amyotrophic lateral sclerosis; ALSFRS‐r, amyotrophic lateral sclerosis functional rating scale revised; DPR, disease progression rate; NA, not available; NFL, neurofilament light chain; pNFH, phosphorylated neurofilament heavy chain.

*p < 0.001, significant difference between the ALS and control groups.

**FIGURE 1:**
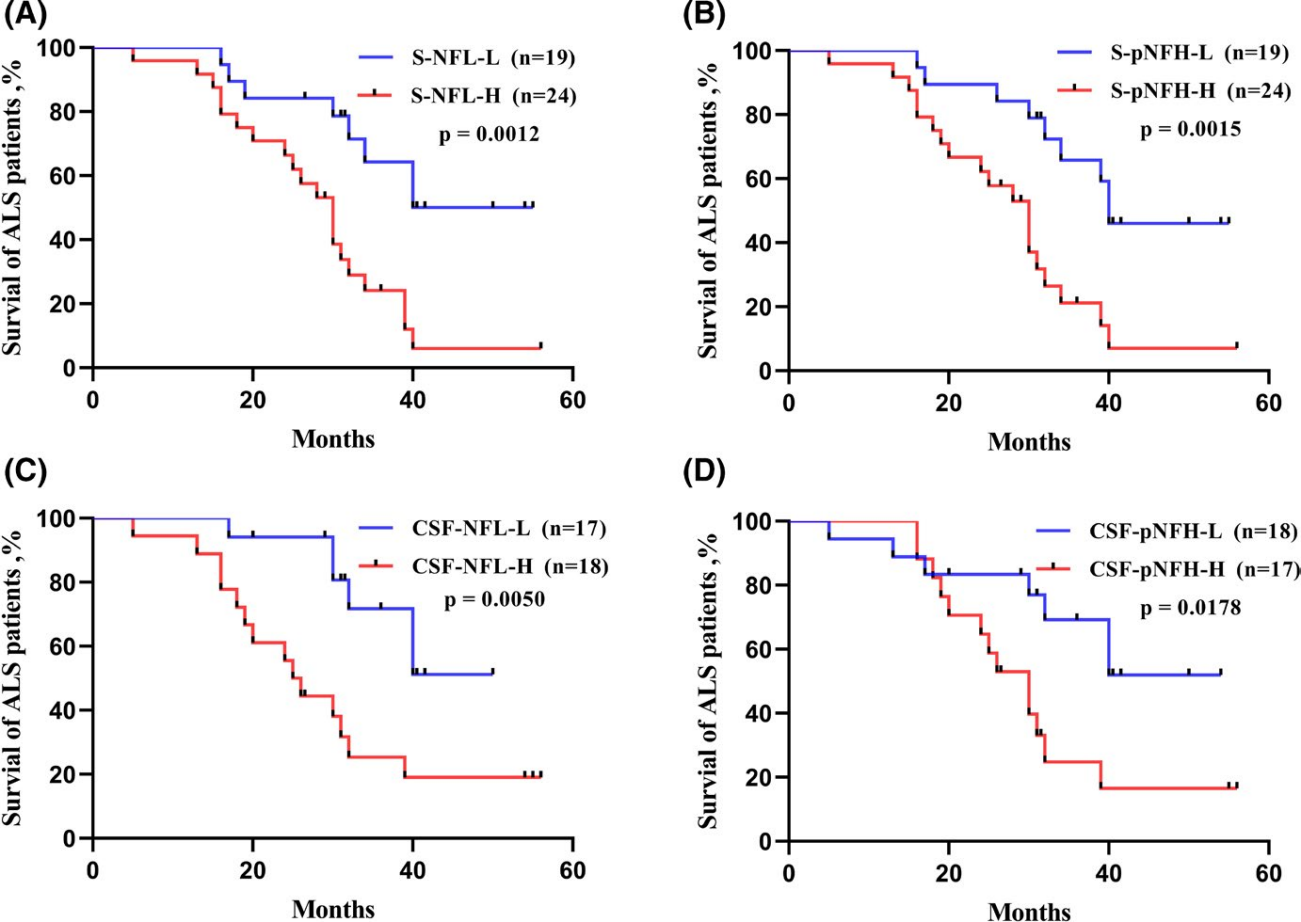
Kaplan‐Meier survival curves. Significant separation of survival of ALS patients was noted when stratified by median levels of NFL and pNFH in serum and CSF samples

43 cases of ALS had been successfully followed up by telephone calls or clinic visits until September 2021. Kaplan‐Meier survival curves showed that survival was significantly shorter for patients with higher serum/CSF NFL or pNFH levels at diagnosis (log‐rank test *p *< 0.0001, Figure 1). Univariate Cox proportional hazards regressions identified S‐NFL, CSF‐NFL, S‐pNFH, CSF‐pNFH, ALSFRS‐r score, DPR, UMN score and cMAP amplitudes as predictors of the mortality hazard ratio in ALS patients among all the tested factors (Table S5). Subsequently, parameters including ALSFRS‐r score, DPR, UMN score and cMAP amplitudes were tested as covariates when serum/CSF NFs were separately introduced in the multivariate Cox models. The results showed that S‐NFL (HR = 3.626, 95%CI: 1.538–8.551, *p *= 0.003) and S‐pNFH (HR = 3.694, 95% CI: 1.569–8.698, *p *= 0.003) were independent predictors of survival. Meanwhile, higher concentrations of CSF‐NFL (HR = 2.999, 95% CI: 0.913–9.850, *p *= 0.040) and CSF‐pNFH (HR = 3.471, 95% CI: 1.030–11.697, *p *= 0.045) were also significantly associated with a reduced survival in ALS.

## 4 DISCUSSION

In this study, we measured levels of NFL and pNFH in the serum and CSF of ALS patients and controls, and explored their feasibility as potential biomarkers in ALS.

We confirmed that levels of NFL and pNFH in the serum and CSF of ALS patients were substantially increased compared with the control group, which was consistent with the results of most previous studies.^2^, ^3^, ^4^, ^5^, ^6^, ^10^ Though we found a negative correlation between baseline NFs levels and ALSFRS‐r, the rates of changes of ALSFRS‐r during follow‐up were not sufficiently available in this study and whether the baseline NFs levels could predict the decline in neuronal functions of ALS patients still needs to be investigated. Similarly, the relationship between NF levels and disease duration observed in the cross‐sectional measurement design is barely due to changes in NFs levels along the natural history of the disease, but rather reflects the fact that aggressive ALS patients with a fast disease progression are referred faster to the neuromuscular centre and investigated with a shorter delay from symptom onset as compared with the slow progressors. As demonstrated in our study and previous research,^11^, ^12^, ^13^, ^14^, ^15^ levels of NFL and pNFH in serum and CSF were positively correlated with DPR, warranting further evidence for the use of pNFH and NFL at an early disease stage as biomarkers for predicting ALS disease progression. The fluctuations of NF levels throughout the entire disease course still need to be further verified with sufficient follow‐up data. A longitudinal study indicated a stable S‐NFL concentration profile after a robust increase S‐NFL level in ALS patients.^16^ This trend is illuminated to be associated with the kinetics of neurofilaments clearance from axons which in turns depends on the rate of MNs degeneration.^17^, ^18^

The positive correlations between CSF and serum NFs levels and UMN score found in this study agree with another study using diffusion tensor imaging that highlighted associations between higher NFL levels and lower DTI fractional anisotropy and increased radial diffusivity in the CSTs of ALS patients^15^ In contrast, we found no associations between NFs levels and LMN biomarkers of axonal injury except a weak correlation between CSF NFL and cMAP amplitudes. Another investigation of CSF NFL in ALS found no association with anatomical extent of clinical UMN involvement, a borderline association with both UMN +LMN involvement and a significant association with the number of regions showing LMN degeneration assessed through EMG.^19^ The relationship between NFs and the varying degrees of UMNs versus LMNs involvement remains a complicated issue.

ROC curves revealed that CSF‐NFL and CSF‐pNFH outperformed S‐NFL and S‐pNFH in discriminating patients with ALS from noninflammatory neurological disorders. However, a positive correlation between NFs in serum and CSF was identified and similar results were unravelled by previous studies.^5^, ^12^, ^19^ Given the convenient and easily accessible ways of serological specimens in contrast with an invasive lumbar puncture for CSF collection, S‐NFs are thereby recommended as valid substitute biomarkers to evaluate the extent of motor neurons degeneration. Additionally, blood‐based measurement facilitates the conduction of a series of longitudinal analyses. With respect to the relatively weak correlation between serum and CSF NFL, there might be several factors. Firstly, ELISA can typically detect neurofilaments in CSF but have undeniably limited sensitivity for detection in blood. Secondly, the possibility of a ‘Hook effect’ due to NFL aggregation in serum samples might cause an underestimation of NFL levels. Thirdly, the full‐length NFL protein was found to be less stable than pNFH^21^ and is consequently more susceptible to proteases degradation. Lastly, the varying degrees of disrupted permeability of blood‐brain barrier and blood‐spinal cord barrier in ALS may lead to different redistribution of NFL, ultimately contributing to various elevated levels of serum NFL. Meanwhile, NFs might find their way to blood via the interstitial fluid of the brain without entering the CSF. It was also proposed that the elevated levels of NFs in serum might be a reflection of the LMN degeneration.^20^ Therefore, the S‐NFL may not accurately reflect the extent of damage to the CNS, leading to the weak correlation between serum and CSF NFL.

Finally, our data suggested that both NFL and pNFH in CSF and serum were strong predictors of survival in ALS patients, even after considering other prognostic factors. This strongly suggests that both NFs have potential roles as prognostic biomarkers of ALS and could theoretically be used to stratify patients in clinical trials. Notably, data for several factors known to influence survival in ALS including nutritional status, respiratory function, gene mutations and frontotemporal dementia were not available, so firm conclusions on independent roles for NFs levels especially in serum samples should be drawn with caution. The main limitation of our work is the small sample size which may not represent the general population. More robust data from larger prospective longitudinal cohorts using regimented protocols and standardized detection methods would be needed to support the application of serum and CSF NFs as reliable biomarkers in clinical practice.

## CONFLICT OF INTEREST

The authors declare no conflict of interest.

## AUTHOR CONTRIBUTIONS

**Jiaying Shi:** Conceptualization (equal); Data curation (equal); Formal analysis (equal); Investigation (equal); Visualization (equal); Writing – original draft (equal). **Xiaohui Qin:** Data curation (equal); Formal analysis (equal); Investigation (equal); Writing – original draft (equal). **Xueli Chang:** Conceptualization (equal); Formal analysis; Investigation; Visualization (equal). **Hong Wang:** Data curation (equal); Investigation (equal). **Junhong Guo:** Funding acquisition (lead); Supervision (equal); Writing – review & editing (equal). **Wei Zhang:** Supervision (equal); Writing – review & editing (equal).

## Supporting information

Fig S1

Fig S2

Fig S3

Fig S4

Fig S5

Fig S6

Table S1‐S5

Supplementary Material

## Data Availability

The data that support the findings of this study are available on request from the corresponding author.
