# Supplementary figures and images for: Neurofilament markers in serum and cerebrospinal fluid of patients with amyotrophic lateral sclerosis
Source: J Cell Mol Med. 2021 Dec 6;26(2):583–7. doi: 10.1111/jcmm.17100 (PMC8743649; doi:10.1111/jcmm.17100)

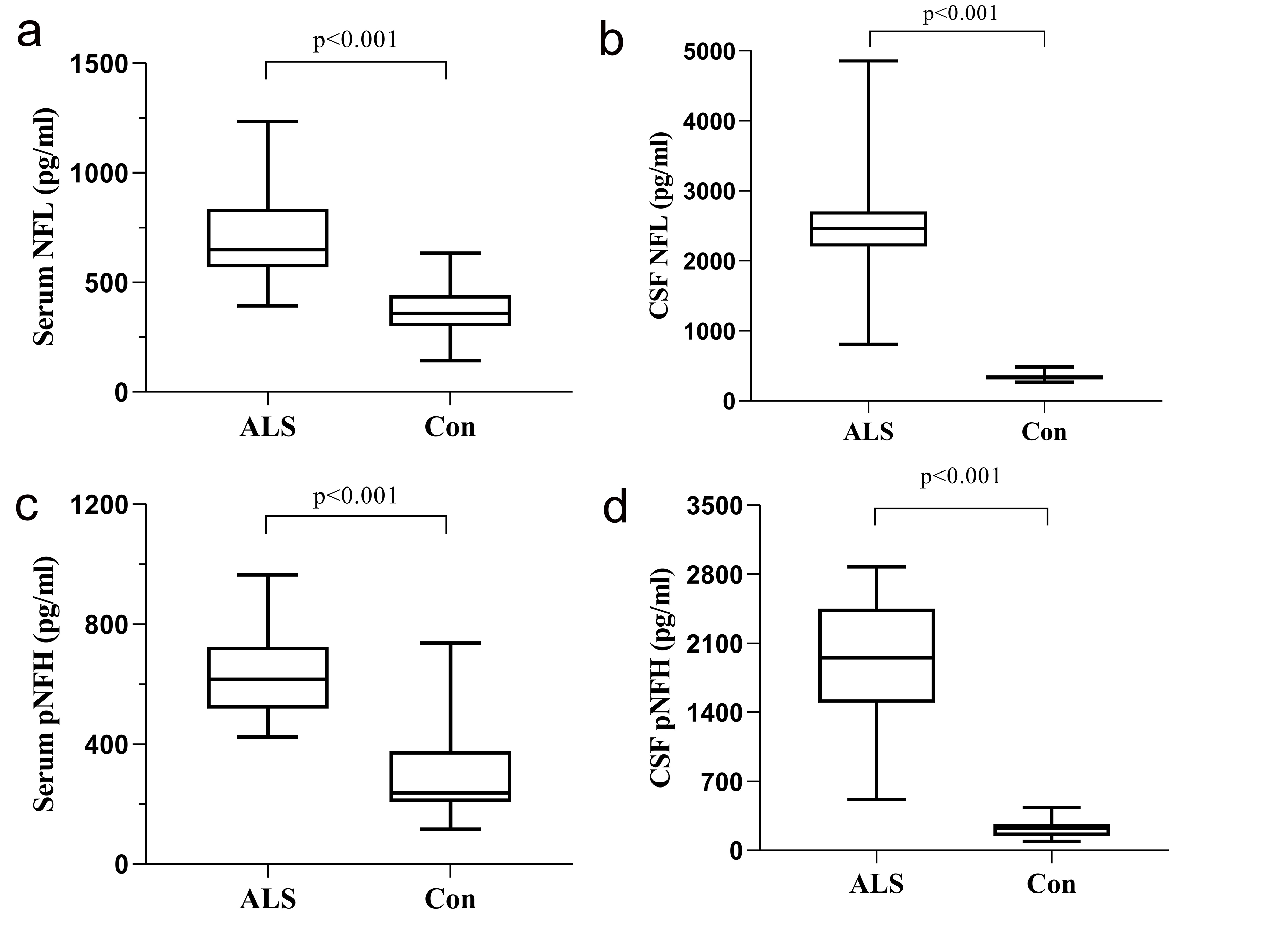

Supplement: Supplementary file 1 — Fig S1 [file JCMM-26-583-s002.jpeg]

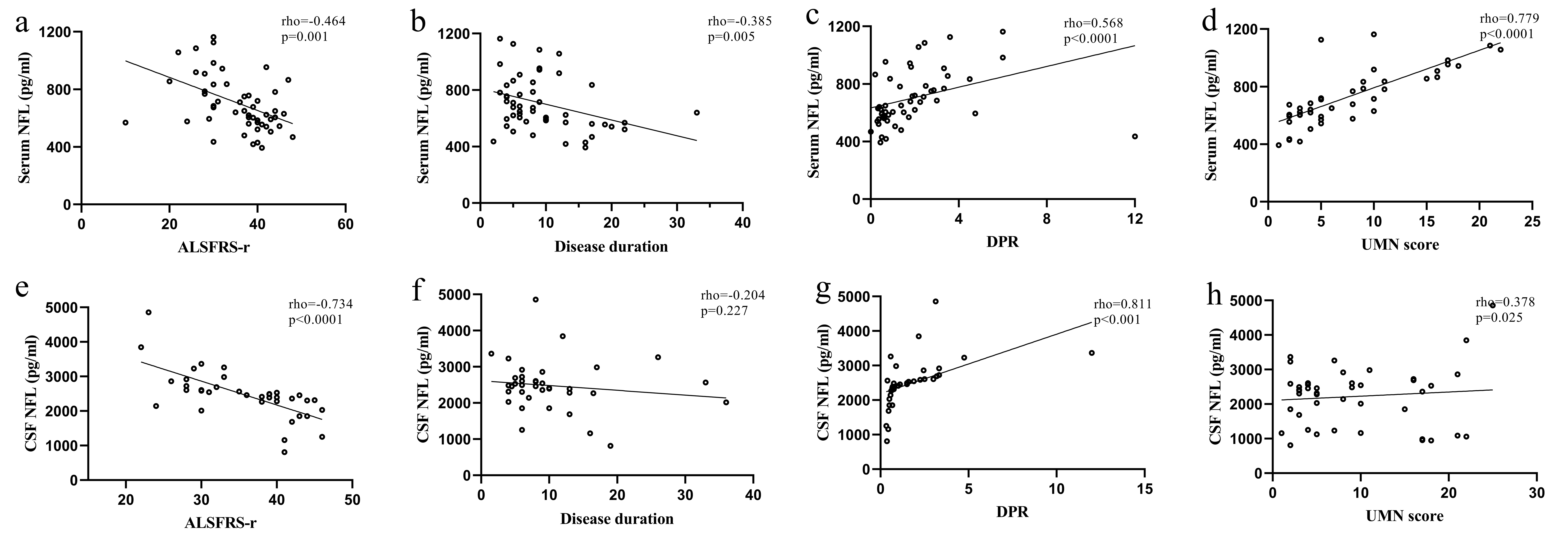

Supplement: Supplementary file 2 — Fig S2 [file JCMM-26-583-s008.jpg]

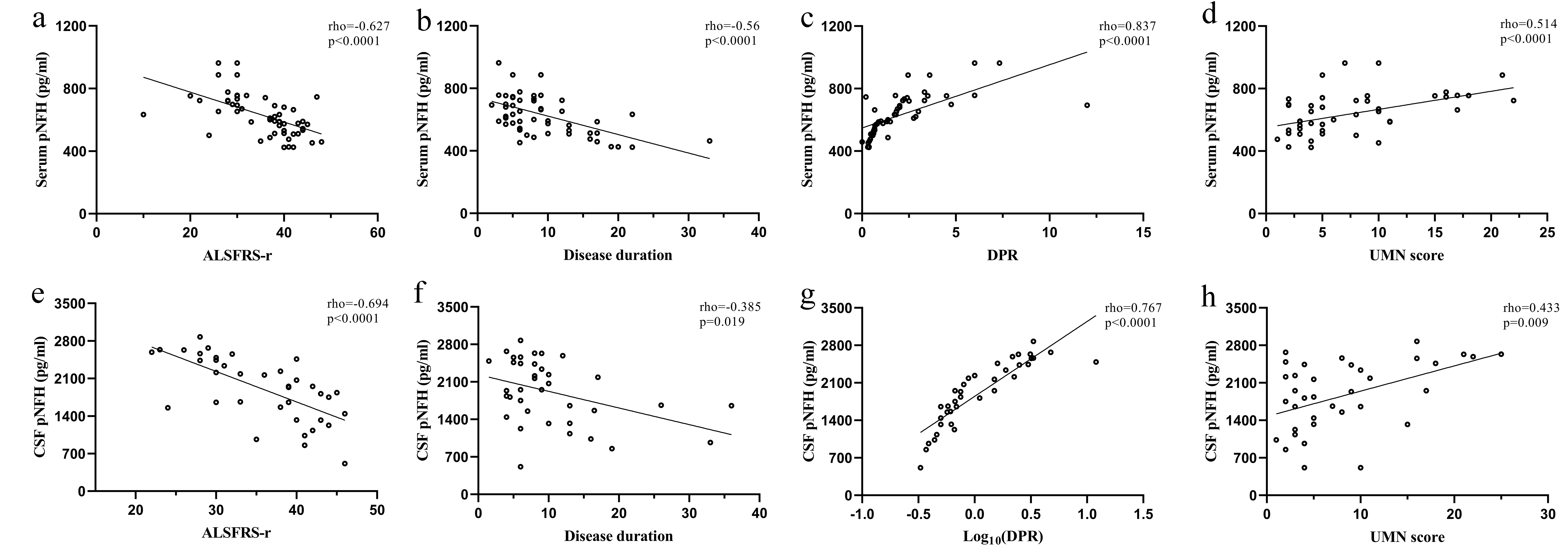

Supplement: Supplementary file 3 — Fig S3 [file JCMM-26-583-s007.jpg]

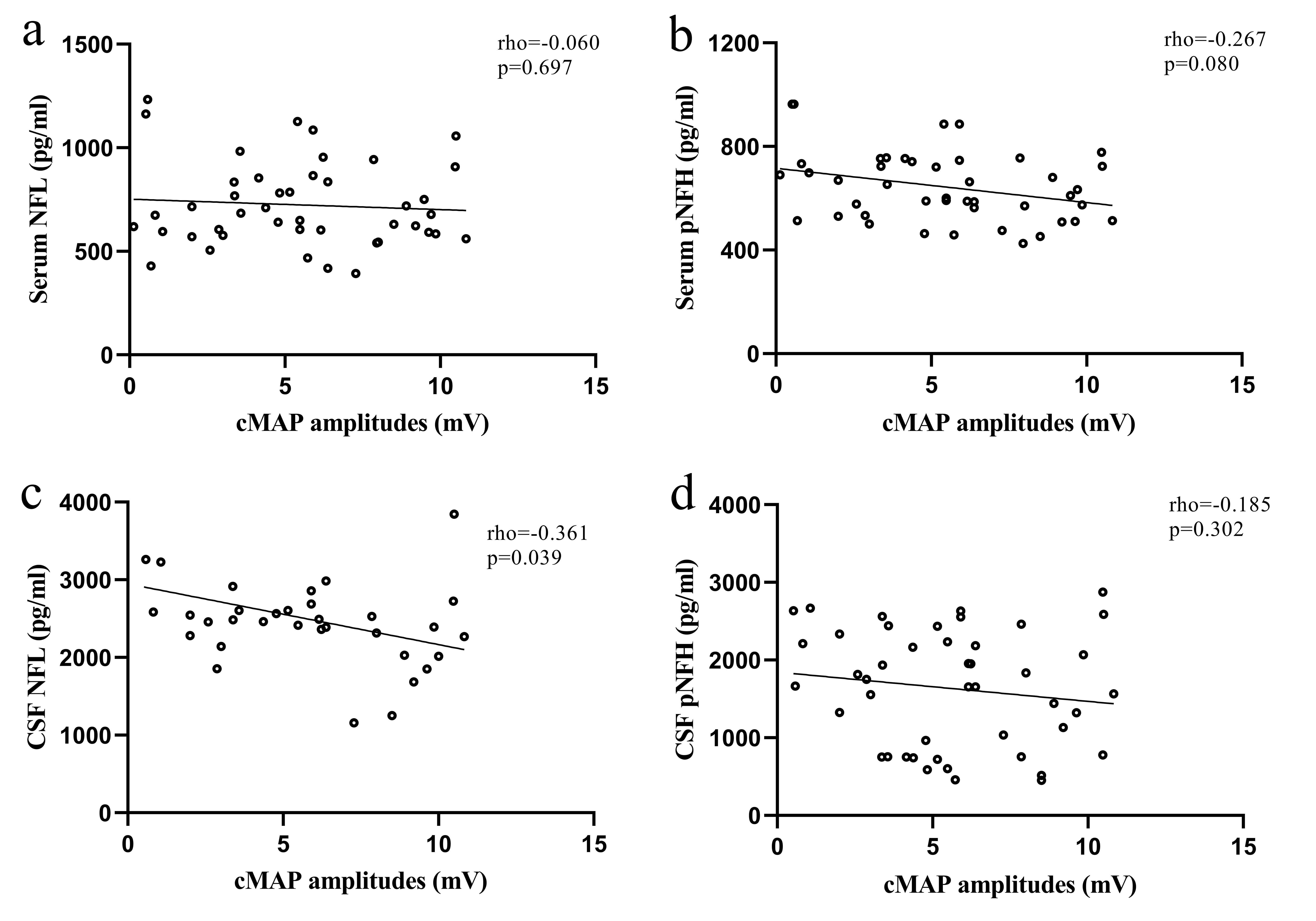

Supplement: Supplementary file 4 — Fig S4 [file JCMM-26-583-s004.jpg]

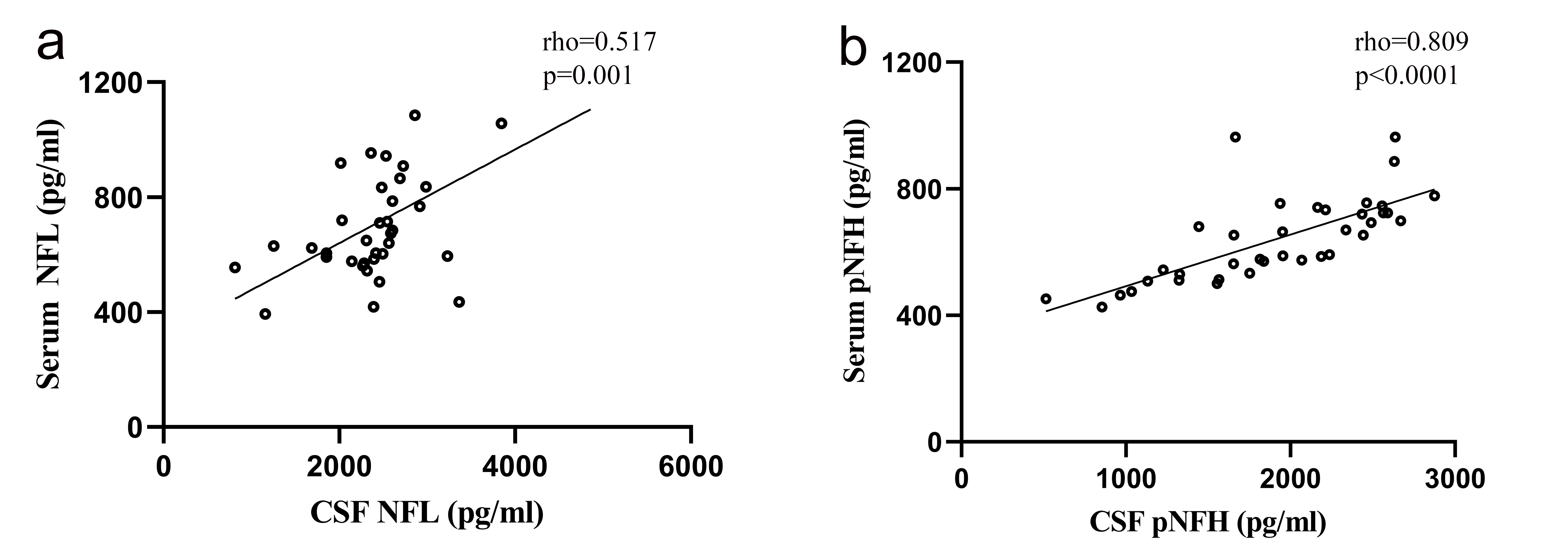

Supplement: Supplementary file 5 — Fig S5 [file JCMM-26-583-s003.jpeg]

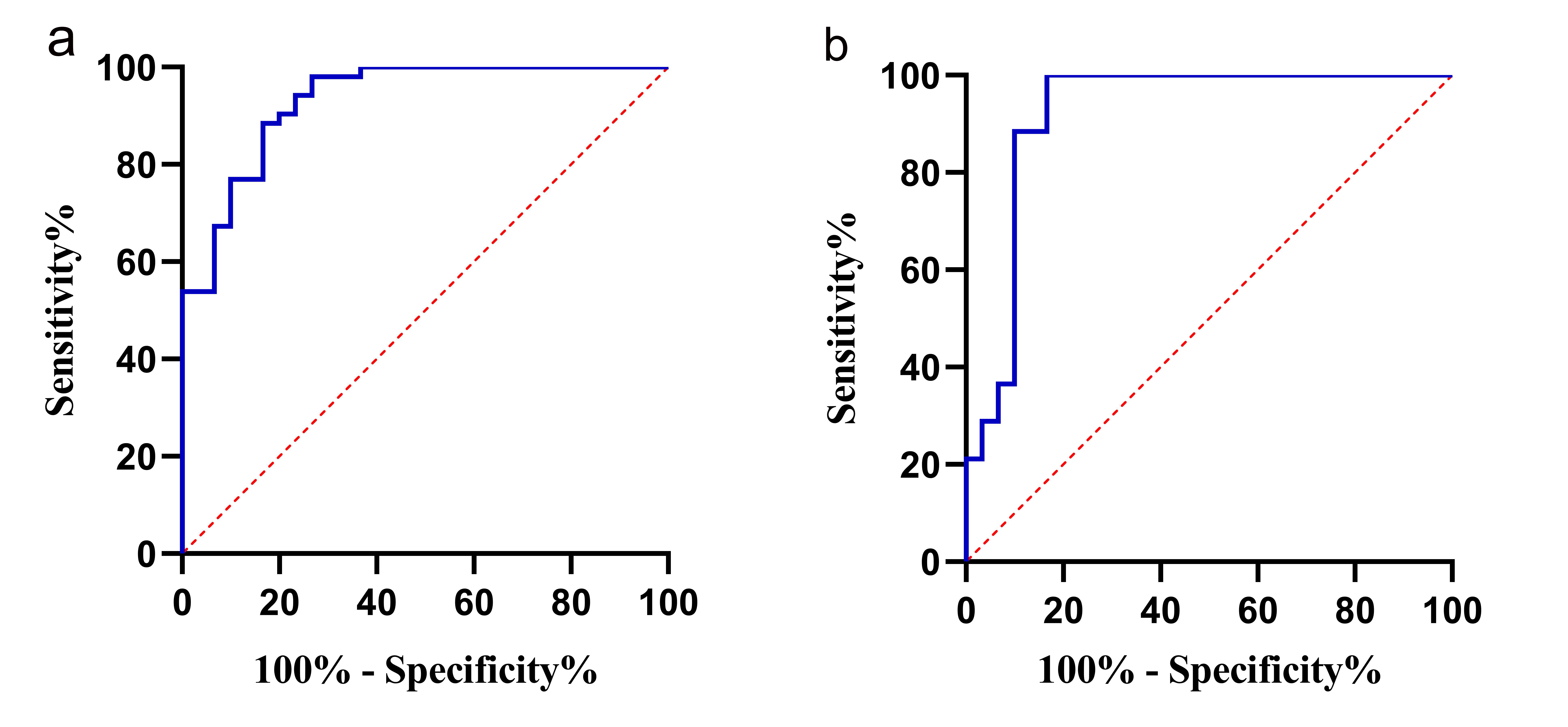

Supplement: Supplementary file 6 — Fig S6 [file JCMM-26-583-s006.jpg]
