# Supplementary material for: Neurofilament markers in serum and cerebrospinal fluid of patients with amyotrophic lateral sclerosis
Source: J Cell Mol Med. 2021 Dec 6;26(2):583–7. doi: 10.1111/jcmm.17100 (PMC8743649; doi:10.1111/jcmm.17100)
Supplement: Supplementary file 7 — Table S1‐S5 [file JCMM-26-583-s005.docx]

Supplementary Table 1: Noninflammatory neurological disorders as controls

| Diagnosis | Number |
| --- | --- |
| Hydrocephalus | n=11 |
| CSF leak | n=2 |
| Intracranial hypotension | n=3 |
| Primary migraine | n=3 |
| Intracranial hypertension | n=4 |
| Hypokalemic paralysis | n=2 |
| Multifocal motor neuropathy | n=2 |
| Non-immune-mediated peripheral neuropathy | n=3 |

Participants were excluded from the study if they had brain trauma, cerebral infarction, or other conditions that alter CSF NFs levels.

Supplementary Table 2: The list of genetic variants in familial or young-onset ALS patients

| Patient | Gender | Age of onset | Diagnosis | Subtype | Variants | Zygosity | Pathogenicity |
| --- | --- | --- | --- | --- | --- | --- | --- |
| 1 | Male | 25 | sALS | Probable | *FUS*,  c.1509_1510delAG  (p.G504Wfs*12) | Het | Likely pathogenic |
| 2 | Male | 50 | fALS | Definite | *NEK1*, c.290G>A  (p.G97D) | Het | VUS |

ALS, amyotrophic lateral sclerosis; sALS, sporadic ALS; fALS, familial ALS; Het, heterozygous; VUS, variants of uncertain significance.

Supplementary Table 3: NFL and pNFH levels in serum and CSF in different subgroups of patients with ALS

| Variable | Site of onset | | Disease duration | | DRP | |
| --- | --- | --- | --- | --- | --- | --- |
|  | Bulbar | Limb | ≤8 months | >8 months | slowly progressive | rapidly progressive |
| S-NFL (pg/ml) | 652.06  (548.14-897.57) | 649.56  (578.41-785.18) | 607.25 (605.06-839.18) | 588.30 (535.97-856.82) | 580.47 (517.01-642.42) | 762.37  (676.78-953.54) |
| P-value | 0.905 | | 0.065 | | **<0.0001** | |
| CSF-NFL (pg/ml) | 2483.34  (2059.59-2707.11) | 2459.04 (2177.05-2795.89) | 2511.16 (2308.28-2715.95) | 2388.96 (1933.05-2711.57) | 2266.76 (1852.41-2388.96) | 2647.01 (2520.14-3046.11) |
| P-value | 0.790 | | 0.209 | | **<0.0001** | |
| S-pNFH (pg/ml) | 648.80  (492.60-743.04) | 605.40  (536.00-697.11) | 685.61 (585.62-747.97) | 546.86 (472.50-656.09) | 521.92 (472.50-579.67) | 721.85 (653.44-755.56) |
| P-value | 0.984 | | **0.004** | | **<0.0001** | |
| CSF-pNFH (pg/ml) | 1952.34  (1444.25-2559.81) | 1884.41 (1470.30-2410.55) | 2188.65 (1768.00-2539.63) | 1655.37 (1227.80-2211.29) | 1553.63 (1132.35-1752.24) | 2452.69 (2205.62-2599.90) |
| P-value | 0.626 | | 0.056 | | **<0.0001** | |

slowly progressive: DPR≤1.44/month in serum; DPR≤ 0.8/month in CSF

rapidly progressive: DPR >1.44/month in serum; DPR > 0.8/month in CSF

Supplementary Table 4: The correlation between the serum/CSF NFs levels and part of ALS clinical parameters

|  | Age | Height | Weight | Gender | Onset site |
| --- | --- | --- | --- | --- | --- |
| S-NFL | r=-0.129, p=0.363 | r=0.286, p=0.220 | r=0.058, p=0.684 | p=0.075 | p=0.905 |
| CSF-NFL | r=0.132, p=0.438 | r=0.174, p=0.304 | r=-0.096, p=0.570 | p=0.588 | p=0.790 |
| S-pNFH | r=-0.093, p=0.512 | r=0.190, p=0.177 | r=-0.037, p=0.797 | p=0.643 | p=0.984 |
| CSF-pNFH | r=0.101, p=0.551 | r=0.079, p=0.641 | r=-0.258, p=0.123 | p=0.328 | p=0.626 |

ALS, amyotrophic lateral sclerosis; NFL, neurofilament light chain; pNFH, phosphorylated neurofilament heavy chain.

Supplementary Table 5: Univariate Cox proportional hazards regression analysis of possible clinical predictors of survival in ALS patients

|  |  | HR (95%CI) | p-value |
| --- | --- | --- | --- |
| S-NFL | S-NFL-L | Ref |  |
|  | S-NFL-H | 3.515 (1.552-7.963) | 0.003 |
| CSF-NFL | CSF-NFL-L | Ref |  |
|  | CSF-NFL-H | 4.218 (1.518-11.721) | 0.006 |
| S-pNFH | S-pNFH-L | Ref |  |
|  | S-pNFH-H | 3.422 (1.506-7.773) | 0.003 |
| CSF-pNFH | CSF-pNFH-L | Ref |  |
|  | CSF-pNFH-H | 4.647 (1.651-13.073) | 0.004 |
| Age | Continuous | 1.166 (0.546-2.491) | 0.682 |
| Gender | Male | Ref |  |
|  | Female | 1.185 (0.549-2.558) | 0.665 |
| Height | Continuous | 1.012 (0.963-1.064) | 0.630 |
| Weight | Continuous | 0.968 (0.930-1.008) | 0.112 |
| Site of onset | Bulbar | Ref |  |
|  | Spinal | 0.884 (0.389-2.008) | 0.768 |
| ALSFRS-r score | Continuous | 0.959（0.915-1.006） | 0.087 |
| Disease duration | ≤8 months | Ref |  |
|  | >8 months | 0.519 (0.220-1.225) | 0.134 |
| DPR | Slow | Ref |  |
|  | Rapid | 2.733 (1.247-5.990) | 0.012 |
| UMN score | Continuous | 1.102 (1.032-1.176) | 0.004 |
| cMAP amplitudes | Continuous | 0.864 (0.744-1.003) | 0.055 |

ALS, amyotrophic lateral sclerosis; NFL, neurofilament light chain; pNFH, phosphorylated neurofilament heavy chain; DPR, disease progression rate; HR, hazard ratio; 95%CI, 95th percentile confidence interval; Ref, reference; cMAP, compound motor action potential.
