# Supplementary material for: Neurofilament markers in serum and cerebrospinal fluid of patients with amyotrophic lateral sclerosis
Source: J Cell Mol Med. 2021 Dec 6;26(2):583–7. doi: 10.1111/jcmm.17100 (PMC8743649; doi:10.1111/jcmm.17100)
Supplement: Supplementary file 8 — Supplementary Material [file JCMM-26-583-s001.docx]

Supplementary Figure 1. Levels of NFs in CSF and serum of ALS group and controls. (a) S-NFL levels were higher in ALS than in controls. (b) CSF NFL levels were higher in ALS than in controls. (c) S-pNFH levels were higher in ALS than in controls. (d) CSF-pNFH levels were higher in ALS than in controls.

Supplementary Figure 2. Correlation between NFL levels and ALS clinical parameters. The S-NFL level was negatively correlated with ALSFRS-r score (rho=-0.464, p=0.001)(a) and disease duration (rho=-0.385, p=0.005)(b), and positively correlated with DPR (rho=0.568, p<0.0001)(c) and UMN score (rho=0.779, p<0.0001)(d). The CSF NFL level was negatively correlated with ALSFRS-r score (rho=-0.734, p<0.0001)(e), but did not correlate with disease duration (rho=-0.204, p=0.227)(f), and positively correlated with DPR (rho=0.811, p<0.0001)(g) and UMN score (rho=0.378, p=0.025)(h).

Supplementary Figure 3. Correlation between pNFH levels and ALS clinical parameters. The S-pNFH level was negatively correlated with ALSFRS-r score (rho=-0.627, p<0.0001)(a) and disease duration (rho=-0.560, p<0.0001)(b), and positively correlated with DPR (rho=0.837, p<0.0001)(c) and UMN score (rho=0.514, p<0.0001)(d). The CSF-pNFH level was negatively correlated with ALSFRS-r score (rho=-0.694, p<0.0001)(e) and disease duration (rho=-0.385, p=0.019)(f), and positively correlated with log would of DPR (rho=0.767, p<0.0001)(g) and UMN score (rho=0.433, p=0.009)(h).

Supplementary Figure 4. Correlation between S-NFs and CSF-NFs in ALS. (a)The S-NFL level was positively correlated with CSF NFL in ALS patients (rho=0.517, p=0.001). (b)The S-pNFH level was positively correlated with CSF-pNFH in ALS patients (rho=0.809, p<0.0001).

Supplementary Figure 5. Receiver operating characteristic (ROC) curves of S-NFL (a) and S-pNFH (b) for discrimination between ALS and disease controls. The corresponding AUCs for S-NFL and S-pNFH were 0.932 (95% CI, 0.878-0.986) and 0.921 (95% CI, 0.843-0.999), respectively.

Supplementary Figure 6. Correlation between NFs levels and cMAP amplitudes. No association was found between S-NFL (rho=-0.060, p=0.697), S-pNFH (rho=-0.267, p=0.080), or CSF-pNFH (rho=-0.185, p=0.302) and cMAP amplitudes except a weak correlation between CSF NFL and cMAP amplitudes (rho=-0.361, p=0.039). The cMAP amplitudes were calculated by averaging bilateral median and ulnar nerve compound muscle action potential (cMAP) amplitudes.
